# Challenges in Automating Extraction of Real-World Radiographic Images and Adverse Events: Lessons From the ICAREdata Initiative

**DOI:** 10.1200/CCI-24-00319

**Published:** 2025-04-18

**Authors:** Steven Piantadosi, Nancy Campbell, Selina Chow, Cassandra Elrahi, Michael V. Knopp, Vaibhav Kumar, Catherine C. Lerro, Donna R. Rivera, Paul G. Kluetz, Andre Quina, Michelle Casagni, Nareesa Mohammed-Rajput, Amye Tevaarwerk, Suzanne George

**Affiliations:** ^1^ Brigham and Women's Hospital, Boston, MA; ^2^ Alliance for Clinical Trials in Oncology Data Innovation Lab, Boston, MA; ^3^ Alliance Protocol Operations Office, University of Chicago, Chicago, IL; ^4^ University of Cincinnati, Cincinnati, OH; ^5^ Oncology Center of Excellence, US Food and Drug Administration, Silver Spring, MD; ^6^ MITRE, Bedford, MA; ^7^ MITRE Corporation, Tampa, FL; ^8^ Mayo Clinic, Rochester, MN; ^9^ Dana-Farber/Harvard Cancer Center, Boston, MA

## Introduction

Real-world data (RWD) and real-world evidence (RWE) reflect usage and effects of medical products.^1^ Electronic health records (EHRs) are used in over 96% of US health care settings and are a primary source for RWD and RWE.^2^ However, RWD is heterogeneously collected, even within the same EHR vendor, reducing its computability and interoperability. Important therapeutic outcomes are often documented in unstructured notes or nonstandardized reports that require manual curation for research, a significant hurdle for RWE.^3^ Complex, variable workflows create additional challenges. The US health care EHR patchwork makes it difficult to produce high-quality research data. Moreover, research data standards are not integrated in EHRs, creating burden, potential for errors, and missing data.^4^ The ICAREdata initiative was designed to generate structured documentation, promoting efficient, low-burden data collection at the point of care in clinical trials.^5^

ICAREdata tests methods of documenting disease status using standardized structured questions during both routine and research visits. The basic approach is to use technologies such as smart forms and smart phrases within the EHR to structure key data with controlled vocabulary at the point of care. Free text is not used. Such data are encoded in the EHR and ultimately transmitted to a research database.^6^ Minimal Common Oncology Data Elements (mCODE) was used to encode key research data.^7^ Then, mCODE-compatible data capture with automatic transmission to external databases used extraction clients and Health Level Seven (HL7) Fast Healthcare Interoperability Resources (FHIR) messaging.^7,8^ Current ICAREdata methods support data standards and interoperability for important assessments such as cancer disease status and treatment plan changes for extraction and analysis.^9^ Efforts described in this paper expanded use into adverse event (AE) and imaging components of the EHR.

## ICAREdata: Building on Previous Infrastructure to Advance RWE

The goal of ICAREdata is to improve automation of RWD extraction and decrease burden on providers and research staff.^5^ EHRs at participating institutions were first configured to deliver data under HL7 FHIR and mCODE standards.^7,10^ Open-source HL7 FHIR standards were used for real-time data extraction. mCODE provided open-source structured responses across all sites, leading to consistency and ease of extraction.^5,7^ Initially, some manual curation was required to extract required information. Feedback surveys indicated that site burden was higher than intended.^5^ Consequently, infrastructure was updated to allow more automated data collection.

For automated capture of end points, aims were to (1) develop and test methods to collect end point–defining image files for patients participating in clinical trials, (2) develop a data model and collection method to identify treatment-emergent adverse events (TEAEs) using data from routine care, and (3) conduct a multi-institutional RWD feasibility study to assess interoperability and completeness of the imaging and AE data collected.

The infrastructure goal was to reduce inefficiencies of current data management procedures in proprietary systems that lack structured data collection and have limited interoperability. For example, if protocol-defined radiographic images could be transmitted for large RWD studies, it would eliminate the need for clinical research associates (CRAs) to manually identify protocol-required image files and upload them to an Imaging Core Lab.

The Alliance for Clinical Trials in Oncology SOLARIS trial (A021703), Vitamin D3 With Chemotherapy and Bevacizumab in Treating Patients With Advanced or Metastatic Colorectal Cancer (SOLARIS; ClinicalTrials.gov identifier: NCT04094688), was identified for evaluation of these aims. This phase III trial was selected on the basis of availability of images, report of required solicited AEs, known side-effect profiles of treatments, and adequate patient enrollment at ICAREdata sites. Patients consented to data collection and extraction as part of SOLARIS trial participation.

## ICAREdata: Results and Infrastructure Development Challenges

Our first aim was to develop and test methods to collect end point–defining image files. The first step was to identify images and elements needed for transmission. Image data elements selected were medical record number, image completion date, ordering provider service, encounter type that generated the order, expectedness on the basis of scheduled protocol time points, order reason, and image source/type (eg, computed tomography, magnetic resonance imaging, and ultrasound). Images were collected according to protocol requirements with a predetermined stop point for data collection to complete this objective (Table 1).

**TABLE 1.** Challenges and Possible Solutions to ICAREdata Infrastructure Development

Research Aim	Steps	Challenge	Potential Solutions
Aim 1: develop and test methods to collect end point–defining image files for patients participating in clinical trials	1. Image file identification	None identified	Not applicable
	2. Centralized network repository for images	VNAs and PACSs require customized solutions to achieve extraction	Development of health IT communication and transmission standards to define interaction between imaging data systems and EHR systems
		They also require additional contracting and permissions, which is an unattainable workflow	
	3. Automated submission of the identified image files	Nonstandardized interactions between EHR and PACS or VNA limits access to image files for extraction Individualized solutions required at each site High provider/CRA burden PHI and deidentification of files and reports process technology issues Infeasible to scale project on the basis of the degree of site-level effort required Inability to scale project on the basis of lack of automation	The certification changes recently proposed by the ONC would require inclusion of imaging hyperlinks in certified EHRs by January 1, 2028; this would enable the viewing or retrieval of images over a network and would expedite this type of work^10^ Re-explore methods with external vendors for access and integration of image files within the EHR system
Aim 2: develop a data model and collection method to identify treatment-emergent adverse events using data collected during routine clinical care	Step 1: definitions of TEAE data elements	None found	Discuss with the scientific and technological community around ways to increase standardization data extraction
	Step 2: develop a TEAE identification tool that would use applied logic to extract necessary data elements	Need to individualized solutions at each site Not scalable and not low burden	
	Site-level query writing and extraction workflows	High site IT burden to write database queries for extraction (140 hours at each site) Efforts at each site are not scalable nor aligned with reducing burden and cost	Potential use of structured reporting for AE identification in EHR to assist with logic Automation of extraction from EHR directly, without site IT-level work for queries being required

Abbreviations: AE, adverse event; CRA, clinical research associate; EHR, electronic health record; IT, information technology; ONC, Office of the National Coordinator for Health IT; PACS, Picture Archiving and Communication System; PHI, protected health information; VNA, vendor neutral archive.

The next step was to assess the capability of creating a centralized, network-level image repository. Because of their size, image files are not stored in the EHR but in other linked systems. Extraction of images files from systems such as vendor neutral archive (VNA) or Picture Archiving and Communication System (PACS) requires access outside of the EHR and may need additional permissions or data linkages from third parties. Like EHR vendor fragmentation, VNAs and PACSs are disjointed, with each requiring customized solutions. Such workflows are unscalable. Adding outside vendors, associated permissions, or contractual agreements makes such work impractical. Protected health information within image files must also be removed before extraction and deidentification. Currently, this redaction is done manually by site CRAs when images are uploaded to the study database. Automating this process to deidentify image files and label them appropriately would reduce site burden and increase efficiency of image transfer. This proved to be infeasible presently.

The third step was to automate submission of image files to a centralized repository. A worklist solution using a customizable query was proposed to guide the export of imaging data. This worklist contains queries to generate required site-level reports that allow identification and extraction of specified images. This worklist query method was infeasible because of required individualized site curation, inability to scale, and limited site resources. It was more time-consuming for staff than the typical method for image transmission because of obstacles around image identification and transmission. Alternative methods for automation, such as new software programs or outside vendor products, were not feasible within the project scope because they were also not scalable. As a result, testing the file collection tool was infeasible.

## Technical Takeaway for Aim 1: Imaging

Substantial infrastructure challenges hamper the ability to obtain source imaging data. Site setup of image files is not standardized. Queries and worklists to retrieve such files require extensive customization as current implementations of EHRs vary widely in how imaging orders, keywords, and procedure descriptions are handled at each site. Therefore, implementations require considerable time, resources, and contractual agreements. Overcoming these challenges requires additional development of health information technology (IT) communication and transmission standards to define interaction between imaging data systems and EHR systems. This could increase scalability and decrease burden. Certification changes recently proposed by the Office of the National Coordinator for Health IT would require inclusion of imaging links in certified EHRs by January 1, 2028.^11^ This would create standardized pointers to enable viewing and retrieval of images over networks and the ability to extract embedded metadata out of digital imaging and communications in medicine image headers and linkage with other EHR-based data. These capabilities will enable and advance this type of work. Once these imaging links are expected components of certified EHRs, they will facilitate advances in clinical decision making, patient care and safety, and efficiency, and drive further innovation in imaging including data sharing, imaging data science, and artificial intelligence-enabled analysis. Improving availability of imaging source data would greatly advance use of RWD to evaluate efficacy. Source imaging data can reduce burden of data acquisition in prospective trials and advance the utility of retrospective RWD. This specific issue may warrant further evaluation to assess feasibility for larger-scale solutions.

Aim 2 was to develop a data model and collection method to identify TEAEs using data collected during routine clinical care. First, TEAE data elements were defined. Solicited AEs are protocol-mandated queries at specified time points. The required solicited AEs chosen had structured data available in the EHR to indicate presence and grade of AE. Queries would be required to extract only those elements that met a prespecified grade.

Four of seven solicited AEs in the SOLARIS protocol were chosen because they had structured signals and logic for extraction on the basis of grade 3 or greater Common Terminology Criteria for Adverse Events (CTCAE) criteria: neutrophil decrease, platelet count decrease, hypertension, and diarrhea.^12^ These represented the known AE profile for treatments used in the trial. Data elements for grading the AEs and extraction were mapped to mCODE and included vital signs, laboratory values, and medication administration. Structured laboratory values for neutrophil or platelet count AEs are already available for extraction from the EHR. Diarrhea, a known side effect of the treatments being used, does not have structured extractable information indicating severity. However, grade 3 or greater might be estimated as a proxy through data elements such as weight loss, dosing modifications, imaging, medications, and potential admissions and/or urgent care visits.

Step 2 was to develop a TEAE identification tool using applied logic to extract necessary data elements. AE definitions and logic identified via CTCAE grading criteria were linked by Logical Observation Identifiers Names and Codes for extraction. This IT linkage was a key strength of the technical process. Definitions for logic applied to extraction of structured data elements associated with a solicited AE term were established on the basis of predefined CTCAE grades. Only those values that met specified grading for the AE criteria would be extracted. For example, only blood pressure (BP) readings that met grade 3 or greater values, systolic BP ≥160 mm Hg or diastolic BP ≥100 mm Hg, would be extracted, thereby avoiding values reflecting grades 0-2.^12^

After identifying AEs and criteria for data elements to extract, we determined that each site would need individual EHR instance-specific queries. The remaining data elements, while not aligned to mCODE Standard for Trial Use v2.0.0, were also structured and extractable in principle via database queries. At the time, natively exposed FHIR-based interfaces were either not available in EHR systems or did not support the required data elements. The Extraction Client served as an interim capability to parse database query results and transform relevant data elements into mCODE-based FHIR. As with the imaging aim, individual query building by sites was not feasible, scalable, or low burden. Although Epic Systems provided EHR vendor services for participating sites, there was not a feasible option for data extraction for our query.

## Technical Takeaway for Aim 2: AEs

In the current state, each site would be required to write database queries for all AE terms as well as definitions and logic to enable accurate and complete data extraction of AEs. Time for all project-related IT work for the four solicited AE terms was estimated to be 140 hours per site. This is more than twice the time required for earlier ICAREdata projects and exceeded site-level resources. Such technical requirements for sites are not feasible or scalable. Sites are also limited by competing demands and internal IT requirements, many of which are clinical care needs. Work required at each site for writing queries and testing a TEAE extraction tool was deemed infeasible. Recognizing site limitations is vital for future work on methods of extraction of these data elements.

In conclusion, reducing burdens of data acquisition for research remains an important objective. Techniques and workflows required are worthy of further research. Although aims of this project were aligned with this objective, we uncovered unanticipated technical challenges, leading to high provider and facility burden and increased costs. Our third aim was therefore not attainable. These foundational challenges stem partly from the fragmented US health care data ecosystem.^13^ Important takeaways include the scalability barriers of proposed workflows along with the degree of customization required at each site. If structured data were collected in routine clinical care, quality of RWD collection would greatly improve.^14,15^ However, solutions to improve data standardization within the EHR must consider human and technical resource constraints. Future work should investigate timely site-level solutions with a focus on resource-sensitive, scalable solutions. Upfront evaluation of the overall data ecosystem, technical hours, and availability of monetary and staffing resources is essential. The Alliance ICAREdata projects will continue to assess methods for improving data collection efficiencies ensuring that they are low burden and increase computability, efficiency, and interoperability of research and RWD for RWE.
